# The disability profile in primary care may depend on the type of care and pain aspects

**DOI:** 10.11606/s1518-8787.2024058005400

**Published:** 2024-12-16

**Authors:** Fernanda de Assis da Costa Ferreira, Angela Baroni de Góes, Raquel Aparecida Casarotto, Tiótrefis Gomes Fernandes, Shamyr Sulyvan de Castro, Ana Carolina Basso Schmitt

**Affiliations:** I Universidade de São Paulo. Faculdade de Medicina. Programa de Pós-Graduação em Ciências da Reabilitação. São Paulo SP, Brasil; II Universidade Federal do Amazonas. Faculdade de Educação Física e Fisioterapia. Manaus AM, Brasil; III Universidade Federal do Ceará. Departamento de Fisioterapia. Fortaleza, CE, Brasil

**Keywords:** Primary Health Care, Family Health, Disability Evaluation, Musculoskeletal Pain

## Abstract

To investigate the relationship between sociodemographic factors, musculoskeletal pain and its characteristics, and the type of primary health care received with self-reported disability.

This is a cross-sectional study, interviewing individuals selected from spontaneous demand for health care in two types of care: health center and family health unit. Disability was investigated using the World Health Organization Disability Assessment Schedule (WHODAS) 2.0 and characteristics of intensity, frequency, duration, number of pain sites, and regions. Measures of association between predictors and disability were performed with non-parametric statistical tests, whereas non-parametric regression models were presented for pain characteristics and for the general population.

In total, 2.3% of family health users and 7.2% of health center users had severe levels of disability. Health center users had more self-reported disability than family health users (p < 0.001). Fewer years of life (p = 0.034) and lower *per capita* income quintile (p = 0.014) were associated with greater disability. The most intense pain and pain in the greatest number of sites increased the disability score by 1.8 (95%CI = 1.0–2.6) and 6.3 (95%CI = 0.1–12.2) points, respectively.

Users who had more disabilities sought care at walk-in health centers, had lower per capita income, presented musculoskeletal pain of worse intensity, and pain in a greater number of sites.

## INTRODUCTION

 Disability is a complex process that goes beyond physical limitations; it is not considered a stable condition and must be widely prevented and treated in primary health care settings ^1^^,^^2^ . The number of chronic diseases lead to higher levels of disability, in addition to resulting in greater use of social and health services and lower quality of life ^3^^,^^4^ . In this context, the assessment of disability must have an approach that is indifferent to the hierarchical order of possible health states based on medical standards, but rather focused on the impact of the disability context on functioning, considering the individual as a whole ^5^^,^^6^ . 

 It is estimated that there are around 978 million people in the world with moderate or severe disability ^7^ . The most recent global estimates suggest that 15.6% to 19.4% of the adult population have experienced some form of disability ^8^ . In the population over 50 years of age, this prevalence ranges from 7.6% to 66.4% in low-income countries ^9^ . 

 Such data may provide a starting point for linking disability to use of services; however, its validity for predicting the need is unknown and may differ with place, time, and person, as the relationships between disability and use of services are bidirectional ^10^ . Thus, health policy makers need to define the priorities for the allocation of resources and, in this way, outline health policies that prevent the onset and worsening of disability within the scope of primary health care ^2^ . 

 The type of health care, clinical aspects such as musculoskeletal pain, and sociodemographic factors may be useful indicators for public policy makers to establish the functioning scenario based on users’ demands. In this sense, the World Health Organization Disability Assessment Schedule (WHODAS) 2.0 instrument absolutely prioritizes the subjective perspective, precisely since it is a model of self-assessment of disability, which makes it advantageous in an environment with a great diversity of comorbidities. It is a simple and easily applicable instrument that may provide a screening for individuals at higher risk of developing more severe disabilities ^11^^,^^12^ . 

This study aims to describe the disability profile of primary care users on spontaneous demand and to verify its association in relation to sociodemographic factors, type of care, and musculoskeletal pain and its characteristics in a region of São Paulo, Brazil.

## METHODOS

 This is a cross-sectional study, in which participants were selected from spontaneous demand in five primary health care units, later grouped into two types of care (health center and family health unit), according to the criterion of having a majority of the reference team in the unit ( Table 1 ). Data collection was conducted in a location with 1,023,486 ^13^ inhabitants, known as the west zone in the city of São Paulo, which is the most populous metropolitan region in Brazil, with a total of 12,252,023 inhabitants ^14^ . 

**Table 1.** Types of assistance care in primary health care.

**Type os assistance care**	**Units**	**Number of people registered**	**Types of teams available**	**Family health Reference team**
Family Health	Mixed (family health + health center)	20,000 registered residents	Family health, medical/nursing, and rehabilitation teams	Partial 60%
	Family Health	15,641 registered residents	Family health and rehabilitation teams	Yes 100%
Health Center	Programmatic	24,766 registered residents	Care teams and professionals linked to teaching (medical/nursing and rehabilitation)	No
	Integrated Health Center	52,369 registered residents and local workers	Family health, medical/nursing and rehabilitation teams	Partial 23%
	Traditional	31,208 registered residents	Health care/nursing teams	No

UBS: unidade básica de saúde.

**Mixed unit:** UBS São Remo; Family health unit: UBS Vila Dalva; Programmatic unit: Butantã School Health Center; Integrated Health Center unit: UBS Jardim Edite; Traditional unit: UBS Caxingui.

Individuals included in the study were aged ≥ 18 years old and able to consent to their participation in writing. The research includes interviews conducted in the waiting room of each health unit. In addition, spontaneous demand was defined, excluding pre-scheduled appointments, collection of clinical exams, simple exchange of prescriptions, withdrawal of exams, and medical reports.

The study was approved by the Research Ethics Committee of the Faculdade de Medicina da Universidade de São Paulo (protocol: 1.781.749) and by the Municipal Department of São Paulo, Brazil (protocol: 1,819,729). All participants signed an informed consent form.

 The sample was systematic, using a sharing procedure proportional to the reference population of each unit. The sample size included 687 people, considering the lowest prevalence of disability in the general population of Brazil (32.8%) ^15^ , with a margin of error of 0.02% in 95% of possible samples and 20% of losses. 

### Dependent Variable

 Disability was investigated using the 36-item version of the World Health Organization Disability Assessment Schedule (WHODAS) 2.0, an instrument directly based on the International Classification of Functioning, Disability, and Health (ICF), whose domains include Cognition, Mobility, Self-care, Getting along, Life Activities, and Participation. The questions concern the difficulties faced by the interviewees over the last 30 days. Scores were assigned to each of the 36 items, being none (1), mild (2), moderate (3), severe (4), and extreme (5), which together resulted in a final score ranging from 0 (no disability) to 100 (maximum disability) ^16^ . These data were categorized based on ICF qualifiers: absent (0–4.9), mild (5.0–24.9), moderate (25.0–49.9), and severe (50.0–95.9). 

 Missing data were handled as suggested in the WHODAS 2.0 ^16^ guidelines, in which the missing item value was replaced by a random value from a similarly matched answered item. 

### Independent Variables

 Independent variables included type of care in primary care (health center and family health unit); sociodemographic data (age in years, gender, education, work, *per capita* income, religion, marital status, type of occupation, and skin color); and pain, defined by the presence of musculoskeletal pain at the time of spontaneous demand, as well as its characteristics such as intensity ^17^ , frequency, duration ^18^ , region ^19^ , and number of pain sites ^20^ . 

### Data Analysis

 Data were analyzed in the Stata statistical package version 16.0. Descriptive statistics were used based on the ICF qualifiers; this sample presented no individuals with extreme/complete disability. In order to characterize the sample by disability levels, the following predictors were used: type of care, gender, age, education, *per capita* income, marital status, work and type of occupation, religion, skin color, and musculoskeletal pain and its characteristics. 

For categorically distributed variables, measures of absolute (n) and relative (%) frequency were presented. Variables distributed continuously were represented as measures of mean and standard deviation (SD) or median and interquartile range (IQR). The prevalence of disability and musculoskeletal pain was estimated with the respective confidence intervals.

 For inferential statistics, the dependent variable was used in its continuous distribution. Nonparametric tests were performed to verify measures of association in the bivariate analyses. Between disability and explanatory variables with more than two categories (categorical age, occupation, education, categorical *per capita* income, religion, frequency, and region and number of pain sites), the Kruskal Wallis and post hoc Dunn test were used. For dichotomous explanatory variables (assistance care, gender, work, marital status, skin color, musculoskeletal pain, and pain duration) the Mann-Whitney U tests were used. Moreover, to verify the relationship between disability and continuous variables (continuous age, continuous *per capita* income, and pain intensity) Spearman’s correlation coefficients were estimated, with results ranging from −1 to 1, in which outcomes were categorized as 0.1 to 0.29 (weak), 0.30 to 0.49 (moderate), and greater than or equal to 0.50 (strong) ^21^ . In the multivariate analysis, the non-parametric Kernel regression model was applied, estimating the weight of the independent variables in the adjusted disability after bivariate analysis (p < 0.20). 

 Regression models were built for the total study sample and according to pain characteristics. For each model, a bootstrap of 1,000 repetitions was used to estimate 95%CI, deriving the mean disability as a function of the explanatory variables. All independent variables were tested for multicollinearity with tolerance for entry into the model, Variance Inflation Factor (VIF) values less than 5 ^22^ . The level of significance was previously set at α = 0.05 and the confidence interval at 95% (CI95%). 

## RESULTS

 Of the 668 individuals who participated in the survey, 498 were from units of the health center type and 170 of the family health type. Table 2 shows that the median score of WHODAS 2.0 was 15.5 (IQR = 5.7–29.2). Minimum and maximum values for age and WHODAS scores were 18 and 91 years and 0 and 82.4 points, respectively. Most participants were women (72.6%), with a mean age of 45.7 years (SD = 16.9) and *per capita* income of 1,155.00 BRL, equivalent to approximately 222.00 USD. Among those with severe disability, 70% were women with a mean age of 46.5 years (SD = 14.6), who sought care on a spontaneous demand in a health center (90%). 

Of the family health unit users, 65.3% (95%CI 57.9–72.0) had some level of disability, whereas, for those who used the health center, the percentage was 80.9% (95%CI 77.2–84.1). Health center users also showed higher prevalence of severe disability, at 7.2% (95%CI 5.6–10.9), compared to the family health type, at 2.3% (95%CI 0.9–5.9).

Regarding the variables associated with disability, family health users had less disability (p < 0.001), as well as Catholics compared to not having a religion (p = 0.0328) and continuous per capita income, which had an inverse relationship with self-reported disability (p = 0.0068). Having musculoskeletal pain was also associated with greater disability (p < 0.001). The overall prevalence of musculoskeletal pain in the study was 59% (95%CI 55.2–62.6).

**Table 2.** Description of sociodemographic and clinical characteristics according to disability (n = 668).

Sociodemographic and clinical characteristics		Disability by level						Linear disability			
		None	Mild	Moderate	Severe	p		Continuous		p	
		n (%)	n (%)	n (%)	n (%)		r	Median	IQR(25–75)		r
**Type of assistance care**	Family health	59 (34.7)	71 (41.8)	36 (21.2)	4 (2.3)	**<** 0.001 *		11.4	2.4 – 23.6	**<** 0.001*	
	Health center	95 (19.1)	233 (46.8)	134 (26.9)	36 (7.2)			16.7	7.4 – 31.1		
**Age (years)**	25 percentile (18–32 years)	30 (18.8)	85 (53.1)	36 (22.5)	9 (5.6)	0.2347**		16.3	8.4 – 26.7	0.0938**	
	25–75 percentile (33–58 years)	83 (25.1)	129 (39.0)	96 (29.0)	23 (6.9)			16.7	4.8 – 31.1		
	75–99 percentile (> 58 years)	40 (24.7)	83 (51.2)	31 (19.2)	8 (4.9)			13.0	5.0 – 24.6		
**Gender**	Mean (SD)	47.5 (15.6)	46.4 (18.0)	44.9 (15.3)	46.5 (14.6)	0.1562***	-0.05	15.5	5.7 – 29.2	0.0614***	-0.07
	Men	47 (25.7)	85 (46.4)	39 (21.3)	12 (6.6)	0.2227*		14.4	4.6 – 26.5	0.2765*	
	Women	107 (22.1)	219 (45.1)	131 (27.0)	28 (5.8)			15.8	5.9 – 29.9		
**Employment**	Unemployed	41 (18.6)	117 (52.9)	51 (23.1)	12 (5.4)	0.7799*		15.1	6.5 – 27.5	0.6473*	
	Employed	113 (25.3)	187 (41.8)	119 (26.6)	28 (6.3)			15.9	4.9 – 30.9		
**Occupation**	Superior members of government and private companies, and science and arts professionals	20 (30.8)	26 (40.0)	16 (24.6)	3 (4.6)	0.7786**		14.0	4.0 – 27.4		
	Mid-level technicians and administrative service workers	11 (17.5)	36 (57.1)	12 (19.1)	4 (6.3)			14.6	8.0 – 25.6		
	Service workers and self-employed	67 (25.0)	111 (41.4)	73 (27.2)	17 (6.4)			16.3	4.9 – 31.1		
	Industry workers, repair, and maintenance	15 (31.9)	12 (25.5)	17 (36.2)	3 (6.4)			17.3	3.2 – 34.7		
**Marital status**	Single	94 (22.0)	200 (46.8)	110 (25.8)	23 (5.4)	0.8396*		15.6	6.0 – 29.1	0.8724*	
	Married	60 (24.9)	104 (43.1)	60 (24.9)	17 (7.1)			15.4	5.0 – 29.4		
**Skin color**	Non-White	96 (22.4)	197 (45.9)	111 (25.9)	25 (5.8)	0.7005*		15.8	6.2 – 29.2	0.5286*	
	White	58 (24.3)	107 (44.7)	59 (24.7)	15 (6.3)			14.4	5.3 – 29.2		
**Education**	Illiterate or less than one year of study	4 (15.4)	12 (46.2)	7 (26.9)	3 (11.5)	0.5102**		15.2	6.7 – 32.1	0.8738**	
	Elementary school or equivalent	54 (24.5)	87 (39.6)	66 (30.0)	13 (5.9)			16.0	5.1 – 31.5		
	High school or equivalent	72 (22.1)	160 (49.1)	75 (23.0)	19 (5.8)			15.4	6.7 – 27.4		
	Higher education/Postgraduate	24 (25.0)	45 (46.9)	22 (22.9)	5 (5.2)			14.4	4.9 – 27.0		
**Per capita income (Reals)**	1st quintile	26 (26.3)	34 ( 34.3 )	33 (33.3)	6 (6.1)	0.1710**		19.5	4.8 – 31.8	0.0727**	
	2nd quintile	18 (17.5)	53 (51.4)	24 (23.3)	8 (7.8)			17.8	7.8 – 30.1		
	3rd quintile	27 (21.9)	61 (49.6 )	31 (25.2)	4 ( 3.3)			14.3	5.3 – 27.6		
	4th quintile	23 (31.1)	33 (44.6)	13 (17.6)	5 (6.7)			11.5	3.3 – 22.8		
	5th quintile	27 (28.7)	46 (49.0)	16 (17.0)	5 (5.3)			13.5	3.7 – 21.9		
	Average (SD)	1.354 (2.073)	1.169 (1.046)	957 (828)	1.015 (763)	0.0197***	-0.10	15.5	5.7 – 29.2	0.0068***	-0.10
**Religion**	Does not have ^a^	18 (16.5)	47 (43.1)	31 (28.5)	13 (11.9)	0.0284**		19.3	9.0 – 36.9	0.0328**	
	Catholic ^b^	95 (26.8)	154 (43.5)	91 (25.7)	14 (4.0)			14.3	4.4 – 27.5		
	Evangelic ^a,b^	31 (21.4)	72 (49.7)	34 (23.4)	8 (5.5)			16.1	5.5 – 26.2		
	Others ^a,b^	10 (16.9)	30 (50.9)	14 (23.7)	5 (8.5)			14.4	8.2 – 36.2		
**Musculoskeletal pain**	No	101 (36.9)	127 (46.3)	43 (15.7)	3 (1.1)	< 0.001*		9.4	1.7 – 19.3	< 0.001*	
	Yes	53 (13.5)	177 (44.9)	127 (32.2)	37 (9.4)			20.5	10.0 – 33.7		
	Prevalence % (95%CI)	23.0% (20.0–26.4)	45.5% (41.8–49.3)	25.4% (22.3–28.9)	6.0% (4.4–8.0)						

SD: standard deviation

n: number of users

95%CI: 95% confidence interval

IQR : interquartile interval

Note: *Per capita* income: 1st quintile (0–475.00 BRL/ 0–91.00 USD); 2nd quintile (476.00–700.00 BRL/ 92.00–134.00 USD); 3rd quintile (701.00–1,000.00 BRL/ 135.00–192.00 USD); 4th quintile (1,001.00–1,500.00 BRL/ 193.00–289.00 USD); 5th quintile (>1,500.00 BRL/ > 289.00 USD). *U mann-Whitney test. **kruskal wallis and Dunn’s post-test. ***Spearman’s correlation. ^a, b^ Equal letters indicate that there was no significance between the groups. Disability levels : none (0–4.9); mild (5.0–24.9); moderate (25.0–49.9); severe (50.0–95.9).

 The mean intensity of musculoskeletal pain in the study was 5.1 (SD = 3.0). Most of the sample had pain only reported in the spine region (37.1%), pain frequency of 6–7 days a week (66%), pain lasting more than 6 months (65.7%), and only one reported pain site (58.4%). Table 3 shows that the most intense and frequent pain in different regions and in a greater number of sites were the characteristics most associated with greater disability. 

**Table 3.** Description of sociodemographic and clinical characteristics in individuals with musculoskeletal pain according to disability (n = 394).

Sociodemographic and clinical characteristics		Disability Continuous		p	
		Median	IQR(25 – 75)		r
**Type of assistance care**	Family health	16.4	5.9 – 27.8	0.0063*	
	Health center	21.8	10.7 – 35.2		
**Age (years)**	25 Percentile (18–32 years) ^a^	21.4	12.5 – 33.7	0.0126**	
	25–75 percentile (33–58 years) ^a^	23.6	10.1 – 35.8		
	75–99 percentile (> 58 years) ^b^	15.3	6.9 – 29.4		
	Continuous	20.6	10.0 – 33.7	0.0089***	−0.13
**Gender**	Men	20.4	11.3 – 29.6	0.7263*	
	Women	20.6	9.3 – 35.0		
**Employment**	Unemployed	16.5	8.1 – 29.9	0.0665*	
	Employed	22.4	10.1 – 36.2		
**Occupation**	Superior members of government and private companies, science and arts professionals	20.6	8.2 – 29.6	0.7263**	
	Mid-level technicians and administrative service workers	22.8	10.0 – 33.6		
	Service workers and self-employed	22.0	10.1 – 36.4		
	Industry workers, repair, and maintenance	29.2	15.6 – 41.5		
**Marital status**	Single	20.6	10.0 – 34.0	0.9075*	
	Married	20.4	10.0 – 33.5		
**Skin color**	Non-White	21.7	10.1 – 33.6	0.4872*	
	White	19.6	9.9 – 35.0		
**Education**	Illiterate or less than 1 year of study	24.2	10.8 – 37.7	0.8043**	
	Elementary school or equivalent	20.6	10.2 – 35.1		
	High school or equivalent	20.5	10.0 – 33.8		
	Higher Education/Postgraduate	20.5	9.0 – 30.5		
**Per capita income (Reals)**	1st quintile	25.2	10.0 – 35.6	0.1200**	
	2nd quintile	22.6	13.8 – 40.1		
	3rd quintile	16.5	9.9 – 29.3		
	4th quintile	14.4	8.2 – 28.5		
	5th quintile	15.9	7.5 – 27.4		
	Continuous	20.6	10.0 – 33.7	0.0587***	-0.11
**Religion**	Does not have	22.8	10.4 – 40.7	0.1273**	
	Catholic	20.3	9.5 – 32.2		
	Evangelic	20.3	9.3 – 32.1		
	Others	21.5	9.2 – 40.1		
**Intensity**	(0–10)	20.6	10.0 – 33.7	< 0.001***	0.38
**Frequency (days per week)**	1–2 ^a^	14.3	6.7 – 27.4	0.0045**	
	3–5 ^a,b^	22.1	11.2 – 31.7		
	6–7 ^b^	22.1	11.2 – 36.9		
**Region**	spíne ^a^	20.2	9.2 – 32.1	0.0001**	
	UL ^b^	10.3	4.2 – 21.2		
	LL ^a,b^	16.0	9.4 – 28.9		
	+ pain regions ^c^	29.0	15.8 – 42.1		
**Number of sites**	1 site ^a^	15.6	6.9 – 27.9	0.0001**	
	2 sites ^b^	25.5	16.3 – 40.5		
	3 or more sites ^a,b^	31.0	15.5 – 44.5		
**Duration (months)**	< 6 months	20.6	9.7 – 36.2	0.8474*	
	> 6 months	20.4	10.0 – 33.5		

n: number of users

IQR: interquartile range

UL: upper limbs

LL: lower limbs.

Note: Per capita income: 1st quintile (0–475.00 BRL/ 0–91.00 USD); 2nd quintile (476.00–700.00 BRL/ 92.00–134.00 USD); 3rd quintile (701.00–1,000.00 BRL/ 135.00–192.00 USD); 4th quintile (1,001.00–1,500.00 BRL/ 193.00–289.00 USD); 5th quintile (> 1,500.00 BRL/ > 289.00 USD). *Mann-Whitney U test. **Kruskal Wallis and Dunn’s post-test. ***Spearman correlation. ^a,b,c^ Equal letters indicate that there was no significance between the groups.

Table 4 presents the results of the multivariate nonparametric regression analysis of the overall sample (n = 668). The variables age, type of care, *per capita* income, religion, and musculoskeletal pain remained in the final model. Predictors explained 15.3% of the variance in this model. The presence of musculoskeletal pain is the strongest variable to attribute greater disability, adding 11.2 points to the WHODAS 2.0 score. Moreover, the type of health care center was also found as a predictor of increased disability, whereas being older and having higher *per capita* income decreased the disability score. 

**Table 4.** Multivariate nonparametric regression estimate between disability and sociodemographic and clinical characteristics (n = 668).

**Sociodemographic and clinical characteristics**		**Disability** r ^2^ = 0.1529	
		Estimated (95%CI)	p
**Type of assistance care**	Family health	**Ref**	
	Health center	5.5 (2.6 to 8.4)	**<** **0.001**
**Age (years)**	Continuous	-0.08 (-0.16 to -0.002)	**0.034**
**Per capita income**	1st quintile	**Ref**	
	2nd quintile	-1.5 (-2.7 to -0.3)	**0.014**
	3rd quintile	-2.4 (-4.6 to -0.4)	**0.019**
	4th quintile	-3.5 (-6.7 to -0.4)	**0.023**
	5th quintile	-5.0 (-9.2 to -0.9)	**0.013**
**Religion**	Does not have	**Ref**	
	Catholic	-2.7 (-7.5 to 1.7)	0.232
	Evangelic	-4.0 (-9.0 to 1.1)	0.115
	Others	-1.4 (-8.4 to 5.2)	0.670
**Musculoskeletal pain**	No	**Ref**	
	Yes	11.2 (8.5 to 13.9)	**<** **0.001**

95%CI: 95% confidence interval

Note: *per capita* income: 1st quintile (0 to 475.00 BRL/ 0 to 91.00 USD); 2nd quintile (476.00 to 700.00 BRL/ 92.00 to 134.00 USD); 3rd quintile (701.00 to 1,000.00 BRL/ 135.00 to 192.00 USD); 4th quintile (1,001.00 to 1,500.00 BRL/ 193.00 to 289.00 USD); 5th quintile ( > 1,500.00 BRL/ > 289.00 USD). Bootstrap for 1,000 reps.

Table 5 presents the results of the multivariate nonparametric regression analysis in the sample with musculoskeletal pain (n = 394). Pain characteristics variables were progressively inserted as a way to verify the best fit for the final model. All variables had VIF < 5, yet regions and number of pain sites presented a strong correlation (r = 0.72) with each other, indicating a possible collinearity between them. The correlation coefficients between the other variables were < 0.38. The choice of permanence of the variable number of pain sites in the final model was due to the lowest p value presented in model 1. Pain intensity was the main characteristic of pain associated with disability, remaining significant in all regression model adjustments. 

**Table 5.** Adjusted multivariate nonparametric regression estimates between disability and pain characteristics variables,. (n = 394).

**Sociodemographic and clinical characteristics**		**Model 1 r ^2^ =0.2443**		**Model 2 r ^2^ =0.6435**		**Final model r ^2^ =0.6386**	
		Estimated (95%CI)	p	Estimated (95%CI)	p	Estimated (95%CI)	p
**Intensity**	(0 – 10)	1.7 (0.9 to 2.4)	**<** **0.001**	1.9 (1.2 to 2.7)	**<** **0.001**	1.8 (1.0 to 2.6)	**<** **0.001**
**Frequency (days per week)**	1 – 2	**Ref**		**Ref**		**Ref**	
	3 – 5	1.2 (-1.3 to 3.9)	0.351	2.3 (-0.7 to 5.0)	0.115	2.4 (-0.6 to 5.2)	0.089
	6 – 7	2.6 (-2.5 to 7.9)	0.325	3.0 (-2.5 to 8.1)	0.276	3.0 (-2.4 to 8.4)	0.267
**Region**	Spine	**Ref**		**Ref**			
	UL	0.1 (-2.1 to 2.2)	0.916	-0.7 (-2.4 to 1.4)	0.483		
	LL	0.3 (-4.2 to 4.5)	0.883	1.2 (-2.1 to 4.9)	0.490		
	+ pain regions	0.6 (-6.1 to 6.9)	0.861	3.8 (-1.4 to 8.8)	0.159		
**Number of sites**	1 site	**Ref**				**Ref**	
	2 sites	2.9 (-0.9 to 6.7)	0.131			4.1 (0.8 to 7.1)	**0.009**
	3 or more sites	5.9 (-1.7 to 13.3)	0.125			6.3 (0.1 to 12.2)	**0.033**

95%CI: 95% confidence interval

UL: upper limbs

LL: lower limbs

Note: Bootstrap for 1,000 reps.

## DISCUSSION

The results of this study suggest that some predictors are related to users’ self-reported disability due to spontaneous demand in primary health care. This research is relevant due to its innovative approach in studying the association between the type of care offered and the disability profile of patients. In the general sample, users of family health units had less self-reported disability compared to those who used a health center. The family health type directs care towards the subject, considering the degree of complexity required. Therefore, having a reference team for the users’ demands, rather than professionals alternating in care, may have been a contributing factor to this finding.

 In the study by Watfe et al. ^2^ , it was found that the predictors being a woman, age ≥ 80 years, ≥ 2 morbidities, and self-perception of poor health status were routinely inserted as warning signs by the family health teams to track disabilities with the possibility of aggravation. In the study by Hustoft et al.² ^3^ , the longitudinality of care was a preponderant factor for individuals to report a lower level of disability in social participation and better self-perception of their health status. Thus, it is expected that effectively coordinated teams have an impact on the continuity of care and that patients experience better care on aspects of functioning when there are relational attitudes from the entire team, as is the case in the family health strategy ^24^ . 

 Regarding the prevalence of disability, 65.3% of family health users had some level of disability, compared to the prevalence of 80.9% in health centers. This finding is similar to that found by Watfe et al. ^2^ , who, in the same city of São Paulo, presented a prevalence of general disability of 56.4% in basic units affiliated to family health. However, it is a value well above the one found in the study by Naidoo et al. ^25^ , which found a prevalence of 38.9% in individuals aged 18 to 64 years with scores above 0 on the continuous scale of the WHODAS 2.0. These differences may be due to the location of the pain site and other context factors, as the latter conducted a cluster survey in households; thus, it is likely that the prevalence of disability is lower in a sample of the general population than in a sample that seeks clinical care ^26^^,^^27^ . Regarding the severe level of disability, this study found a prevalence of 6.0% in the general sample, 7.2% in health centers, and 2.3% in family health. In comparison with other studies, disability in a more general context was verified in the study by Salinas-Rodríguez ^1^ , which found 8.0% of severe disability in older adults from low- and middle-income countries. In the context of samples with specific conditions, Karami et al. ^28^ evaluated individuals with physical and intellectual disabilities and presented 28.9% of severe disability in their study. It is likely that those with more severe disabilities are less likely to participate in studies in a broader context ^29^ . 

 With regard to the prevalence of musculoskeletal pain, 59.0% reported having pain at the time of seeking care in primary health care units. This study presented an association between musculoskeletal pain and disability, so that answering “yes” to the presence of musculoskeletal pain increased the continuous score of the WHODAS 2.0 by 11.2 points. The positive association between pain and self-reported disability has been discussed in several articles ^30^^-^^32^ . Although this relationship is not always observed in a proportional way, a functional improvement may be found without monitoring the pain reduction and vice versa ^33^^,^^34^ . 

 In the multivariate evaluation of pain characteristics, only the intensity and number of pain sites remained significant in the final model. Despite the understanding that the characteristics of greater pain severity (worse intensity, more frequent, in different regions and more sites) increase disability, when these are analyzed together, intensity becomes the main expression associated with the individual’s disability. Pain intensity is a prominent component in the assessment of chronic pain, although people’s tendency to overestimate pain when using this measure must be considered ^35^ . Silva et al. ^36^ reported in their study that pain intensity, general and localized, had greater correlations with WHODAS 2.0 scores than other characteristics. For the authors, greater comprehensiveness of care, opposing fragmentation, can be attributed to the management of intensity, in the understanding that intervening in the reduction of global pain intensity is a better strategy than managing it in specific locations. 

 In addition to intensity, the number of pain sites was also relevant in this analysis. The dose-response effect with incapacity has also been found in some studies ^12^^,^^20^^,^^36^ indicating that multiple pain sites should be given greater attention in care to prevent greater severity of incapacity. 

*Per capita* income was an important predictor of self-reported disability. This corroborates a previous study in which WHODAS 2.0 scores were higher for lower-income participants ^37^ . Similar results were also found by Waterhouse et al. ^38^ , who found that the poorest income quintile was associated with severe disability and the number of chronic diseases reported. In general, individuals with generalized disability are more likely to occupy positions of low socioeconomic status, including unemployed or employed with low pay, having a lower educational level, and lower family income ^39^ . 

 Age was also a factor associated with disability, so that being younger decreased the disability score when the multivariate regression model was analyzed, although this difference was not significant (p > 0.05) in the bivariate analysis of the general sample. These results diverged in most studies that assessed disability in older adults ^29^^,^^37^^,^^38^ . However, a possible explanation is that, in the primary health care setting, older individuals with more severe difficulties sought the units by spontaneous demand less than those individuals who were younger with the same degrees of perceived difficulty, which could suggest a worse access for older adults with higher levels of disability. 

 In general, care and access to health must be guaranteed by the different types of care in primary health care and health teams must adjust to the most frequent demands with strategies with greater impact, dealing with phenomena of functioning, dependence, independence, illness, and health, while adhering to the main guidelines on the biopsychosocial model of health ^40^ . 

## STUDY IMPLICATIONS AND LIMITATIONS

The results suggest that the subjects’ lower report of disability is indicative of better longitudinal care with the health service, so that the units that mostly have the family health reference team may provide greater surveillance of the conditions that most contribute to functional deterioration in their territory. In addition, understanding the characteristics of pain in this population can be useful to define assertive approaches to pain care that promote an improvement in disability and quality of life. Future studies can explore the relationship of assisted care as a causal factor for the functioning profile in a broader population.

This study shows some limitations. First, it does not fully explore the disability profile based on the type of care in primary health care, as it was necessary for users to go to the collection units. Thus, it is possible that users with more severe disabilities were not interviewed. Another issue is memory bias, so that the participants, in addition to reporting the intensity of pain at the time, were also asked to report it during the crisis, which did not always coincide with the pain the user had at the time of the interview. Finally, in this study, we did not verify the comorbidities of users in spontaneous demand, neither to account for them nor qualitatively classify them as possible predictors associated with disability. It is possible that these data could outline a better scenario of the profile of users who most seek care in primary health care, considering the health conditions that most interfere with self-report of disability.

### Highlights

Health center users have more disabilities than family health unit users

Musculoskeletal pain is an important predictor of disability

Pain intensity and site are associated with worse levels of functioning

## CONCLUSION

 Users of health centers, with lower *per capita* income, with fewer years of life and with the presence of musculoskeletal pain had more self-reported disability. Among those with musculoskeletal pain, it was found that pain of a more intense nature and in a greater number of sites in the body was associated with worse severity in the continuous disability score. We highlight that the assistance care of primary health care was an important predictor of the level of disability.
